# Synthetic Glyconanoparticles Modulate Innate Immunity but Not the Complement System

**DOI:** 10.1021/acsabm.2c00026

**Published:** 2022-04-18

**Authors:** Chandradhish Ghosh, Patricia Priegue, Harin Leelayuwapan, Felix F. Fuchsberger, Christoph Rademacher, Peter H. Seeberger

**Affiliations:** † Department of Biomolecular Systems, Max Planck Institute of Colloids and Interfaces, Am Mühlenberg 1, 14476 Potsdam, Germany; ‡ Institute of Chemistry and Biochemistry, Freie Universität Berlin, Arnimallee 22, 14195 Berlin, Germany

**Keywords:** gold nanoparticles, oligomannans, innate immunity, immunomodulators, adjuvants

## Abstract

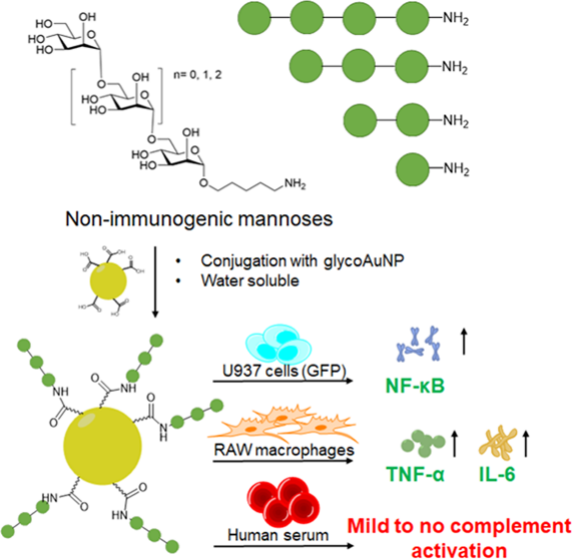

Nanoparticles that modulate innate immunity can act as vaccine adjuvants and antigen carriers and are promising alternatives to conventional anticancer therapy. Nanoparticles might, upon contact with serum, activate the complement system that might in turn result in clearance and allergic reactions. Herein, we report that ultrasmall glyconanoparticles decorated with nonimmunogenic α-(1–6)-oligomannans trigger an innate immune response without drastically affecting the complement system. These negatively charged glyconanoparticles (10–15 nm) are stable in water and secrete proinflammatory cytokines from macrophages via the NF-κB signaling pathway. The glyconanoparticles can be used as immunomodulators for monotherapy or in combination with drugs and vaccines.

## 1 Introduction

Cancer immunotherapy is a promising alternative to chemotherapy that is plagued by toxicity and resistance.^1,2^ Immunotherapy aims at exploiting the patient′s immune system to combat tumors. Approved therapies include molecular therapy (e.g., IL-2), cellular therapy (e.g., T-cell therapy), vaccines, and antibodies.^3^ Adaptive immune effectors are impaired by immunosuppression around the tumor microenvironment.^4^ Agents that enhance innate immunity may overcome these challenges either in combination with other drugs or as monotherapy and in addition, may serve as adjuvants and antigen carriers in vaccines.^3,5^ The development of novel innate immunomodulators is required to address these issues.^6^

The body’s immune response to an invading pathogen offers inspiration for the design of novel immunomodulators. Glycans displayed on the surface of pathogens but absent in humans are recognized by host-cell receptors leading to innate and adaptive immune responses.^7^ Thus, mimicking these glycan-protein interactions can be harnessed for designing therapeutics including glycoconjugate vaccines, antibody-drug-conjugates, antigen delivery systems, and immunomodulators.^8−15^ Since nature uses multivalency in its interactions, a common strategy to develop immunomodulators employs a multivalent display of sugars such as galactofuranose, alpha-galactose, α-fucosyl-β-alanyl amide, and oligomannoses on proteins and nanoparticles.^12,16−18^ None of the systems that target adaptive immunity have been tested for complement activation, considering that rapid clearance would greatly limit their efficacy. Complement activation may further lead to unwanted inflammation, allergy, and anaphylaxis.^19,20^ There is no clear understanding of the minimum number of sugar units required and the role of the size and charge of the nanoparticle in eliciting an innate immune response without activating the complement system. Answers to such questions will aid the design of the next generation of innate immunomodulators.

The identification of sugars that are mildly proinflammatory but not antigenic is key. The cell-wall capsules of pathogens such as *Candida albicans*, *Mycobacterium tuberculosis,* and *Leishmania mexicana* contain polymannoses with α-(1–2), α-(1–3), α-(1–6), β-(1–2), and β-(1–4) linkages.^21−23^ While multivalent formulations of some branched oligomannoses have been investigated as anti-infective agents against viruses, parasites, and fungi, α-(1–6) branched oligomannosides are rarely studied and show mild proinflammatory properties.^24−29^ In an effort to exploit the properties of such sugars toward the development of novel innate immunomodulators, we report here the synthesis and biological activity of short α-(1–6) oligomannoses in the context of monovalent and multivalent systems. As multivalent carriers of the sugars, we used water-soluble glycosylated-gold nanoparticles (2 nm) that are reduced and stabilized by thioglucoses.^30,31^ These glyconanoparticles were then evaluated for their ability to trigger NF-κB signaling, secrete proinflammatory cytokines from RAW macrophages, and activate the complement system. This study underlines the potential of the glycol–gold nanoparticles for use as adjuvants and vaccine carriers.

## 2 Results and Discussion

### 2.1 Design and Synthesis

**Figure 1:**
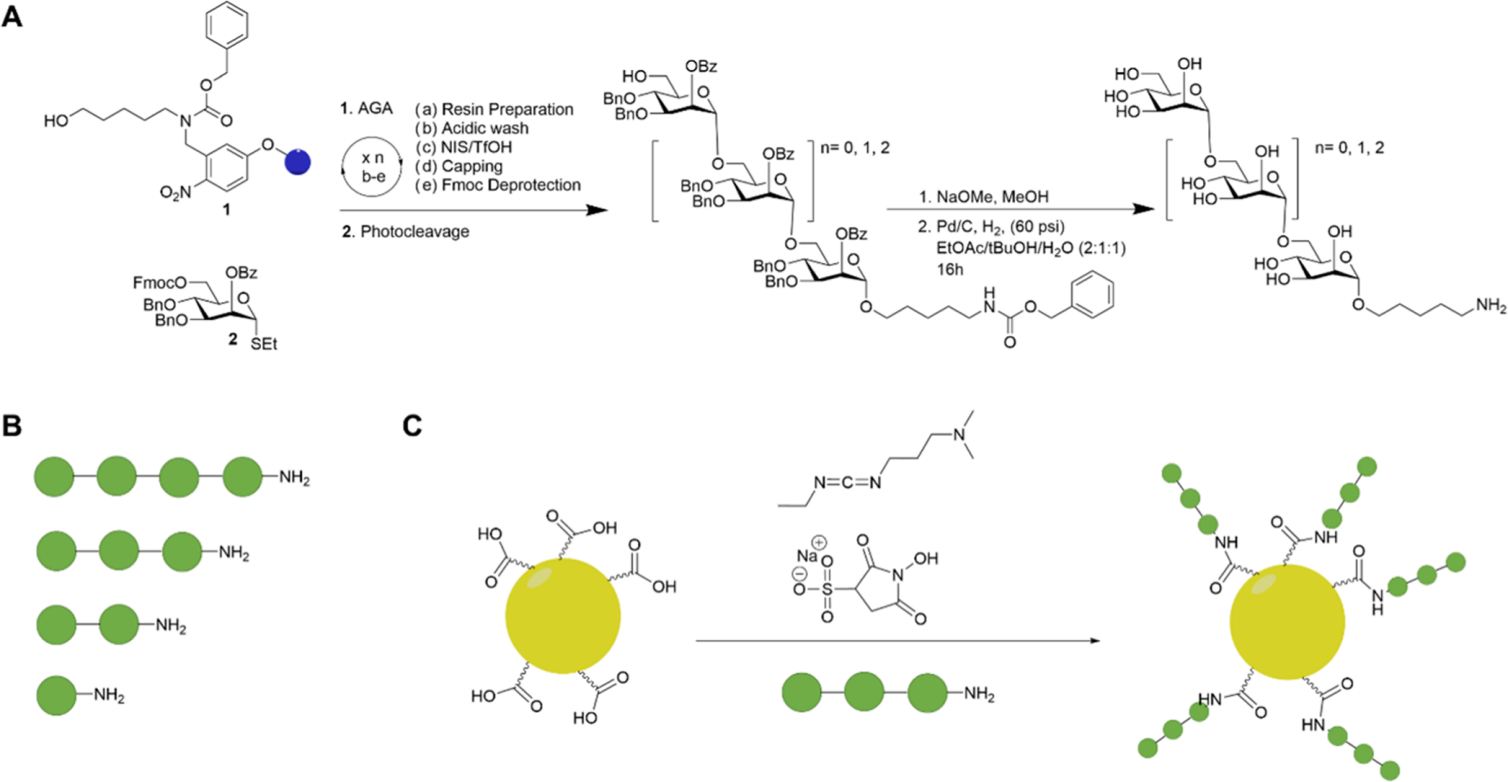
(A) Scheme for the synthesis of mannose oligomers using automated glycan assembly followed by global deprotection. (B) Pictorial representation of mannose α-(1–6)-oligomers used in the study. Every filled green circle represents 1 unit of mannose. (C) Decoration of trimers of mannose α-(1–6) on glycogold nanoparticles using EDC/sulfo-NHS chemistry.

The oligomannans were prepared by automated glycan assembly (AGA) using a homebuilt instrument and following established protocols (Figure 1).^32−35^ For AGA synthesis, Merrifield resin modified with a photocleavable aminopentanol linker (**1**, 40 mg) was placed in a reaction vessel and washed with TMSOTf in CH_2_Cl_2_ at −20 °C for 3 min (wash module) to remove any residual base and water. Then, the mannose building block **2** (6.5 equiv) was delivered to the reactor, followed by the activating solution (NIS/TfOH in CH_2_Cl_2_/dioxane, −20 °C for 5 min and then 0 °C for 20 min). Any unreacted hydroxyl groups were acetylated using the capping module (MsOH in Ac_2_O/CH_2_Cl_2_, 20 min) before the C-6 hydroxyl group of the growing chain was exposed by applying piperidine (20% in DMF) for 5 min. The coupling cycle was repeated until the desired oligomers were assembled. At the end of the glycan synthesis, the oligomers were cleaved from the solid support using a continuous flow photoreactor. The crude oligomers were purified by high-performance liquid chromatography (HPLC) before removal of benzoyl protecting groups via methanolysis with sodium methoxide, followed by hydrogenation using palladium on charcoal. The unprotected sugars were purified again by HPLC and fully characterized before conjugation to gold nanoparticles.

Gold nanoparticles were prepared by reducing and capping aurochloric acid with thioglucose.^31^ The thioglucose is partially oxidized during the process to yield carboxylic acid groups that can be used for further functionalization. The purified oligomannosides were attached to the AuNPs by reacting the amine of the linker at the glycan-reducing terminus, and the carboxylic acid groups of glycogold nanoparticles were activated with 1-ethyl-3-(3-dimethylaminopropyl)carbodiimide (EDC) and *N*-hydroxysulfosuccinimide (sulfo-NHS). Then, a solution of the respective mannose oligomers was added and sonicated (for 2 h) to expedite the process and prevent aggregation (Figure 1A). The resulting solution was dialyzed in MilliQ water overnight to obtain the (mannose)*_n_*-conjugated nanoparticles (Man*_n_*@AuNPs) that were further purified by passing them through a 0.45 μm filter. This solution was characterized using ultraviolet absorption (UV), infrared spectroscopy (IR), dynamic light scattering (DLS), transmission electron microscopy (TEM), and atomic force microscopy (AFM).

### 2.2 Characterization

**Figure 2:**
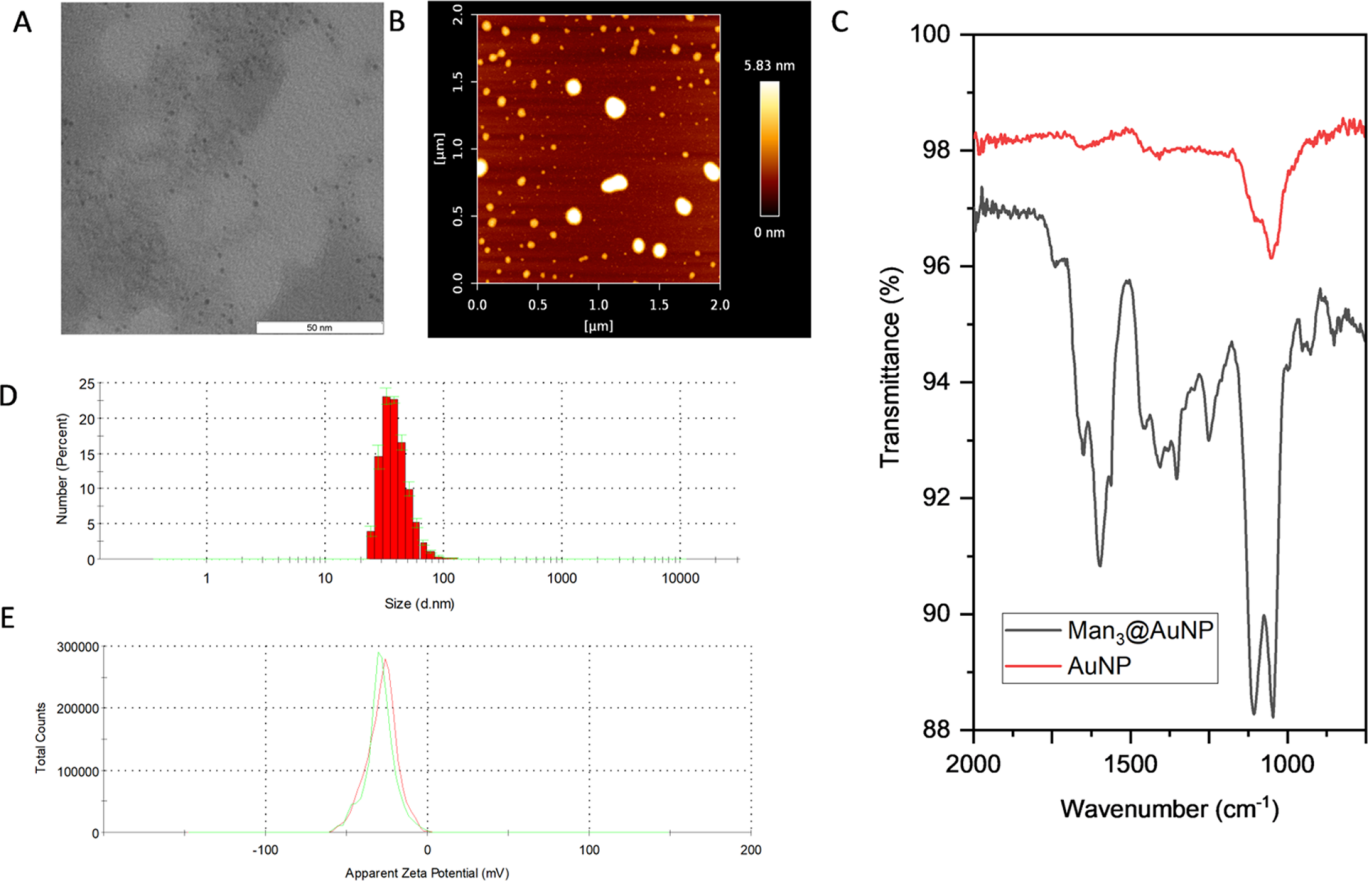
Characterization of Man_3_@AuNPs as a representative nanoparticle. (A) TEM images of Man_3_@AuNPs show the core structure of the gold nanoparticles to be <5 nm (scale bar 50 nm). (B) AFM image of Man_3_@AuNPs. (C) IR spectra of Man_3_@AuNPs show prominent amide bond peaks at 1654 cm^–1^ and 1574 cm^–1^ that are absent in the case of AuNPs. (D) Hydrodynamic radius of Man_3_@AuNPs. (E) Zeta potential of Man_3_@AuNPs is −30 mV.

The size of the gold clusters in Man*_n_*@AuNPs was determined to be smaller than 5 nm according to TEM analysis (Figure 2A and Supporting Information). Atomic force microscopy was used to determine the actual particle size (Figure 2B). AFM images reveal that all of the particles tend to cluster (Supporting Information). The sizes of clusters and the single particles have been measured. The height of the single sugar-conjugated nanoparticles from an AFM scan revealed a variation in the size centered around 10 nm (Supporting Information), while that of the unconjugated AuNPs was 2–3 nm. The oligomer length had no drastic impact on the nanoparticle size (Supporting Information). Amide bond formation was evident from distinct peaks at 1654 cm^–1^ (carbonyl stretching) and 1574 cm^–1^ (N–H bending) observed in the IR spectra of all conjugated nanoparticles (Figure 2C and Supporting Information). The nanoparticles had hydrodynamic radii of ∼40 nm (number percent, Figure 2D) and no significant difference was noted in going from a monomer to a tetramer. The clustering of the nanoparticles possibly contributes to higher hydrodynamic radii. They bore a surface negative charge and were moderately stable in water as evident from a ζ potential of −30 mV (Figure 2E and Supporting Information). Next, the classical anthrone-based method was employed to quantify the concentration of the sugar present in the suspension. Anthrone forms a pale green complex upon reaction with acid-hydrolyzed carbohydrates that absorbs at 630 nm. A standard curve was created by titrating a known concentration of mannose with anthrone (Figure S5). Then, the nanoparticle solution was treated with the same reagent and the absorbance was correlated with the standard curve to obtain the exact concentration of the sugar (Table S1). Inductively coupled plasma atomic emission spectroscopy was used to quantify the gold concentration in the sample (Table S1). These purified nanoparticles were then studied for their immunological activity. For simplicity, we used the gold concentration as a reference for all further studies, and samples were diluted in biological media to contain 1 μg gold per mL solution. Higher concentrations were not used as uncontrolled immune activation is detrimental to the body as explained below. All samples were evaluated for the presence of contaminants such as LPS using the LAL assay and were found to be much lower than 0.5 EU mL^–1^.

### 2.3 Binding to Concanavalin A

To ascertain whether the mannoses retain their ability to bind to mannose binding proteins after conjugation to the gold nanoparticles, we checked the ability of the compounds to bind to concanavalin A. Mannan was used as a positive control and PBS was used as a negative control. All Man*_n_*@AuNPs bound to concanavalin A (Figure S6). The binding efficacy of the naked AuNPs was comparable to that of the negative control PBS. Having established the lectin binding efficacy of the nanoparticles, we checked their ability to activate the cells of the innate immune system *in vitro*.

### 2.4 Induction of NF-κB

**Figure 3:**
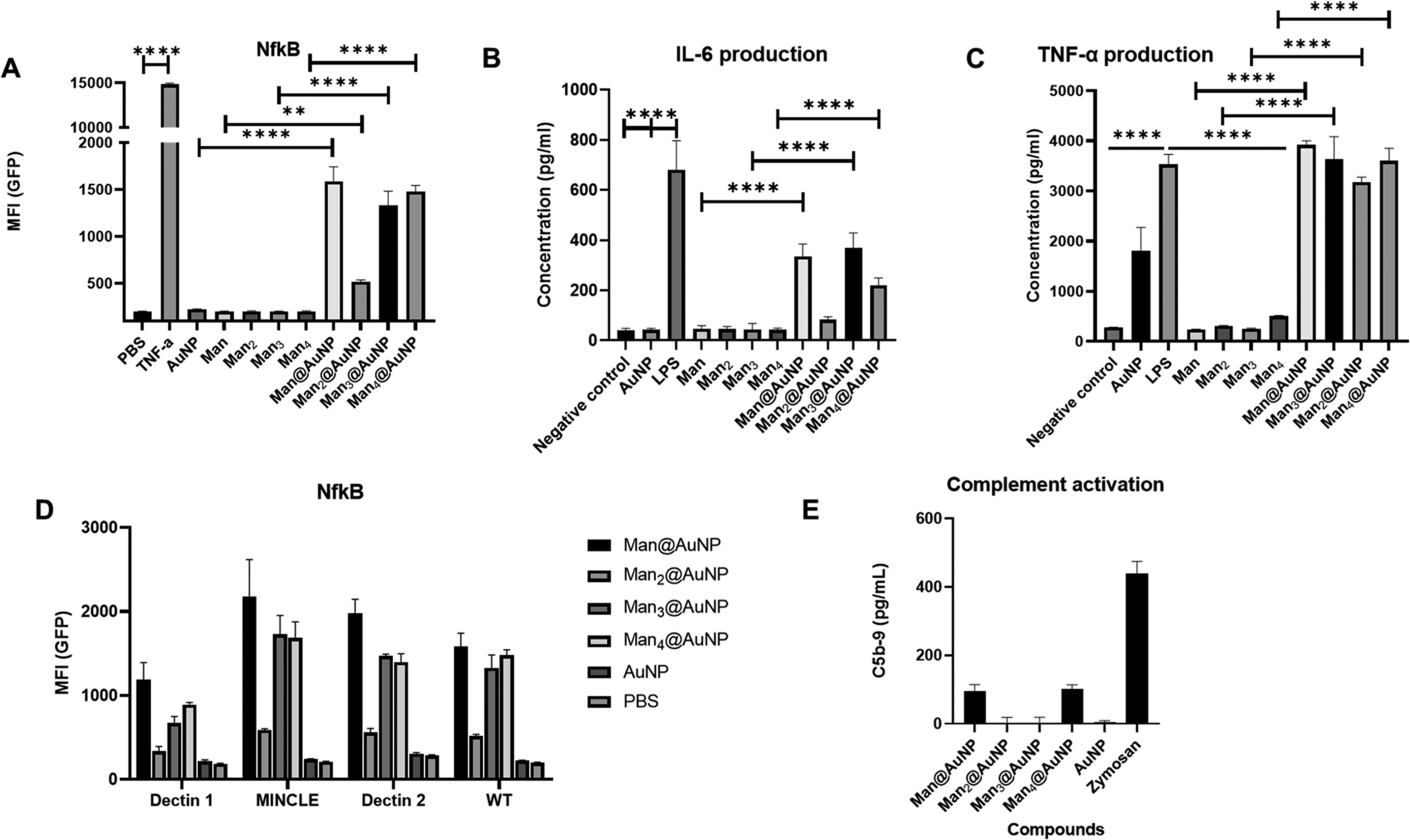
(A) Monocytic U937 cells were transduced with a lentivirus encoding NF-κB-driven GFP expression quantified using flow cytometry. PBS is used as a negative control and TNF-α as a positive control. All mannose-conjugated nanoparticles induce NF-κB activation unlike the unconjugated AuNPs and oligomers of mannose. (B) IL-6 secretion in RAW macrophages after overnight incubation with compounds as measured by ELISA. LPS is used as a positive control. (C) TNF-α secretion in RAW macrophages after overnight incubation with compounds as measured by ELISA. LPS is used as a positive control. (D) NF-κB activation is not dectin-1, dectin-2, or MINCLE pathway-dependent. The lectin overexpressing cells show no increase in NF-κB activation in comparison to the wild-type U937 cells upon treatment with the nanoparticles. (E) ELISA-based detection of SC5b-9 (terminal protein of complement activation) in human serum treated with the compounds. The effect of PBS (negative control) was subtracted from the data (no significant difference noted). Each data point represents the mean standard deviation of at least duplicate experiments. *p* values of <0.05 were considered statistically significant. *, *p* < 0.05; **, *p* < 0.01;***, *p* < 0.001; and ****, *p* < 0.0001.

Immune cell activation was first determined by checking the ability of the compounds to activate NF-κB signaling. The transcription factor NF-κB is a vital mediator of inflammatory responses and plays an important role in both adaptive and innate immunity. It induces the expression of various proinflammatory genes, including those encoding cytokines and chemokines.^36^ Compound-mediated induction was studied using monocytic U937 cells with an NF-κB reporter system. The U937 cells were transduced with a lentivirus encoding NF-κB-driven GFP expression for quantification by flow cytometry. The cells were incubated with different compounds in the same concentrations (1 μg mL^–1^) to study the activation of NF-κB. Tumor necrosis factor-α (TNF- α) was used (100 ng mL^–1^) as a positive control while PBS was used as a negative control. None of the monovalent sugars or unconjugated gold nanoparticles were able to enhance GFP expression (Figure 3A). In comparison, all mannosylated nanoparticles activated NF-κB signaling, albeit the intensity was much lower than that of TNF- α. The dimer-conjugated AuNP was the least effective in NF-κB induction, but there was no significant difference between the efficacies of the other nanoparticles (Figure 3A).

### 2.5 Upregulation of Proinflammatory Cytokines

The effect of the compounds on immune cells such as RAW macrophages was studied. Specifically, the release of proinflammatory cytokines such as TNF-α and IL-6 upon treatment was evaluated. Both TNF-α and IL-6 have a variety of roles in immunity, with the former having anticancer implications as well.^37,38^ RAW 264.7 macrophages were first treated with the compounds and lipopolysaccharides (LPS, positive control) and then their supernatant was analyzed for the presence of cytokines using ELISA. The unconjugated monomer, dimer, trimer, and tetramer of α-(1–6)-linked mannose oligomers elicit no response in macrophages (Figure 3B,C). Plain gold nanoparticles trigger some TNF-α but no IL-6 response in cells. Mannose-conjugated nanoparticles at concentrations of 1 μg mL^–1^ result in more cytokine respective unconjugated controls. Although LPS induced more IL-6 secretion than the gold nanoparticles, the effect was similar in the case of TNF-α. In fact, it was observed that a higher concentration of the compounds elicited a higher response (data not shown). Due to the detrimental effects of uncontrolled response, the concentrations were kept at 1 μg mL^–1^. Secretion of TNF-α was not dependent on the length of oligomannose while some variation was noticed in the secretion of IL-6 (Figure 3B,C).

### 2.6 Mechanism of Action

The immunomodulatory effect of the nanoparticles may be the result of interactions with C-type lectins present on the macrophages that trigger immunological pathways against pathogen-associated molecular patterns like mannoses present on microbial cell surfaces.^13^ In this respect, DC-SIGN is a well-studied mannose receptor. Instead, we probed interactions with other common receptors expressed on macrophages that are responsible for antifungal immunity such as dectin-1, dectin-2, and MINCLE (macrophage inducible Ca^2+^-dependent lectin receptor).^39^ We overexpressed dectin-1, dectin-2, and MINCLE on U937 cells and probed for enhancement in NF-κB activation. None of the lectin overexpressing cells showed an increase compared to the wild-type U937 cells (Figure 3D). The oligomannose-containing nanoparticles do not preferentially activate NF-κB via these receptors.

### 2.7 Complement Activation

Complement activation by nanoparticles might lead to opsonization that precludes their intended action.^40^ Although sometimes beneficial in prophylactic protection, uncontrolled complement activation is harmful and contributes to disease progression.^41^ In cancer therapeutics, complement activation by nanoparticles has several implications and gold nanoparticles are also known to interact with the complement system.^42−44^ To be used as an immunotherapeutic these glyconanoparticles must not induce complement-mediated anaphylaxis. Thus, we sought to study the effect of the mannosylated glycogold nanoparticles of the complement system. A nanoparticle-mediated rise in the serum concentration of C4d (a recognized marker of both classical and lectin pathways) and SC5b-9 (a marker of the terminal pathway of the complement cascade) levels is indicative of complement activation. The rise in the concentration of the two proteins upon incubation of the nanoparticles (1 μg mL^–1^) with human serum for 30 min was measured. In comparison to the negative control (the effect of the PBS was deducted from the levels of each compound), no significant elevation in levels of SC5b-9 was detected in human serum upon treatment with any of the gold nanoparticles (Figure 3E). The nanoparticles were not able to increase the C4d levels significantly above their respective background in any case (data not shown). Thus, it was inferred that mannosylated nanoparticles do not trigger complement activation and possess only proinflammatory properties at this concentration, paving the way for their development as adjuvants and antigen carriers. These results accord with what has been shown in a study where shorter mannose oligomers are not able to bind mannose-binding lectin (MBL) and therefore not able to activate the MBL pathway of the complement system.^45^

### 2.8 Toxicity of the Nanoparticles

Finally, we evaluated the toxicity of the nanoparticles to check for harmful side effects. As can be seen in Figure S7, none of the mannose-conjugated nanoparticles were toxic to the macrophages, while Triton X completely lysed the cells. This opens up the possibility of using the nanoparticles as an immunotherapeutic in animal models of infection and cancer in future.

## 3 Conclusions

In an effort to develop innate immunotherapeutics, we showed that a multivalent presentation of nonimmunogenic fragments of α-(1–6)-oligomannans on ultrasmall nanoparticles imparts immunomodulatory properties to the formulation. NF-κB activation was much higher in the conjugates in comparison to the naked nanoparticle, but the efficacy did not have a trend with respect to the size of oligomers used. Macrophages upon treatment with these nanoparticles secreted higher levels of proinflammatory cytokines but activated the complement system only mildly (if at all) in human serum. Although these nanoparticles hold promise as innate immunomodulators, overactivation must be controlled. Thus, the nanoparticles can act as adjuvants to vaccines and chemotherapeutics, without the detrimental effects of nanoparticle-mediated complement activation.

## 4 Experimental Section

### 4.1 Synthesis of Oligomannosides

The automated glycan assembly of oligomannosides was performed using previously reported procedures.^34,35^ Nuclear magnetic resonance (NMR) and high-resolution mass spectrometry (HRMS) spectra of the compounds obtained were in complete agreement with the previous report and used for further synthesis.^46^

### 4.2 Synthesis of Gold Nanoparticles

The gold nanoparticles were prepared using a previously published protocol.^48^ In a representative synthesis, 1-thio-β-d-glucose sodium (Glc-SNa) (500 μL, 41.2 mM) was added to HAuCl_4_ (6.25 mL, 2.89 mM) at room temperature. Instantly, a change in the color from yellow to brown was observed, which indicated the formation of the gold nanoclusters. This suspension was vortexed for 5 min until the color turned dark brown. The solution was then transferred to a Falcon tube with a filter and centrifuged at 3000*g* for 30 min. This procedure was repeated three times and finally, the residue was diluted with more water. UV visible spectra of the resultant solution were measured, and the number of AuNPs was calculated using a previously reported protocol.^31^

### 4.3 Synthesis of Oligomannose-Conjugated Gold Nanoparticles

AuNPs (1 mL, 0.35 μmol) were mixed with 1 mL of PBS and then diluted with 5 mL of water. To the solution, 1-ethyl-3-(3-dimethylaminopropyl) carbodiimide (EDC, 3.5 μmols) and *N*-hydroxysulfosuccinimide (sulfo-NHS, 3.5 μmols) were added and sonicated. Then, to this solution, the oligomannoses (3.5 μmols) obtained from automated glycan assembly were added and the mixture was sonicated further for 2 h at room temperature. The contents were dialyzed (tubing diameter 4.6 mm, MWCO 6-8 KD) in 1.5 L of water overnight.

### 4.4 Infrared Spectroscopy

lR measurements were performed using a Perkin Elmer Spectrum 100 Fourier transform infrared (FTIR) spectrometer. Aqueous dispersions of glyconanoparticles were first lyophilized and then resuspended in 20 μL of methanol. The methanolic suspensions (5 μL) were dropped on the probe to dry before applying a pressure gauge to record the infrared spectrum. The transmittance spectra furnished in the main text were baseline corrected and slightly smoothed for presentation.

### 4.5 Transmission Electron Microscopy

TEM measurements were performed on a Zeiss EM 912 Omega. The samples were prepared by gently dropping the samples (20 μL) onto grids and subsequent solvent evaporation in a dust-protected atmosphere.

### 4.6 Dynamic Light Scattering (DLS)

DLS measurements were carried out at a scattering angle of 173° with a Malvern Zeta Nanosizer working at a 4 mW He–Ne laser (633 nm). The nanoparticles were all measured in MilliQ water. The refractive index chosen was for gold, and the solvent chosen was water. Every measurement was carried out three times with 10–100 counts each (automated). Several samples from several different syntheses were measured. The average size (by number) remains similar. In the text, the representative image of one such experiment is presented.

### 4.7 Zeta Potential Measurement

A Malvern Zetasizer instrument was used to measure the electrophoretic mobility of nanoparticles at different times of dialysis against MilliQ water. The Helmholtz–Smoluchowski equation was used to correlate the measured electrophoretic mobilities with the ζ potentials. Three replicates of each sample were measured six times at 25 °C in MilliQ water.

### 4.8 AFM Characterization and Analysis

Samples were prepared on freshly cleaved mica and dried at room temperature. AFM images were acquired using a commercial AFM system (JPK NanoWizard 3 and 4). Measurements were performed in AC mode with SNL-10 probes (Bruker) at 25 °C, 35–40% RH. AFM images were collected with 1024 × 1024 pixels/frame. Each AFM tip was characterized prior to usage. Analyses of AFM images were performed with JPK Data Processing software. Note that for the height analyses of the AFM images, the baseline height was leveled against the flat base plane of the substrate. All AFM images were only subjected to the primary first order flattening correction to remove sample tilt so that potential artifacts induced by other image processing steps were avoided as much as possible.

### 4.9 Inductively Coupled Plasma Optical Emission Spectrometer (ICP-OES) to Determine Gold Concentrations

The concentration of gold in the solution of nanoparticles was measured using an inductively coupled plasma optical emission spectrometer (ICP-OES) (Optima 8000; Perkin Elmer, Massachusetts). For this, an external calibration series from 0.1 to 5 mg L^–1^ was prepared using a gold standard solution. Sample solutions were first dried (typically ranging from 200 μL to 1 mL) and dissolved in aqua regia; this solution was analyzed using ICP-OES. Each experiment was done in triplicate and the experiments were repeated at least twice. The mean value (in μg mL^–1^) of at least two independent experiments was reported.

### 4.10 Sugar Quantification

Anthrone reacts with hexoses in the presence of sulfuric acid to form a colored compound that can be detected by absorption spectroscopy. The reaction is concentration-dependent and can be monitored by measuring the absorbance at 620 nm. A freshly prepared solution of anthrone (0.5%, w/w) in sulfuric acid was added slowly to stock solutions (0.5 mL) of mannose at different concentrations to create a standard curve. The resulting solutions were gently mixed and heated to 80 °C for 10 min. The absorption was recorded at 620 nm to make the calibration curve (Supporting Information, Figure S5). The same procedure was repeated with the glyconanoparticles and the absorbance was recorded. The absorbance was then correlated with the calibration curve to obtain the amount of sugar present in the solution.

### 4.11 Concanavalin A Binding ELISA

Costar plates were coated with 50 μL of the different compounds (1 μg mL^–1^ in coating buffer) and controls and incubated overnight at 4 °C. The wells were washed twice with TMS buffer (20 mM tris(hydroxymethyl)aminomethane (Tris)–HCl, pH 8.0; 150 mM NaCl; 1 mM CaCl_2_; 2 mM MgCl_2_) and blocked with 100 μL of TMS with 1% BSA for 30 min at room temperature. After one wash with PBS (200 μL/well), the plate was incubated at 37 °C with 50 μL of fluorescein concanavalin A (Con A) (3 μg mL^–1^) in BSA-TMS for 1 h. The wells were washed four times with TMS (200 μL) and incubated at room temperature with 50 μL of goat-anti FITC HRP (1:2500 dilution) in BSA-TMS for 1 h. After four washes with TMS (200 μL), 100 μL of substrate solution (TMB and H_2_O_2_) was added, and after some minutes at room temperature, the reaction was stopped with 50 μL of H_2_SO_4_. Finally, the plate was read at 450 nm with an ELISA reader. The experiment was repeated twice and in triplicates.

### 4.12 Reporter Cell Assay

U937 GFP NF-κB reporter cells (made with Cignal reporter lentivirus, Qiagen, The Netherlands) were used in the log phase, 100 μL were plated in a 96-well plate with 3E4 cells per well. Lectins were expressed using lentiviral transduction, as described earlier.^47^ Cells were challenged in complete media (RPMI with 10% FBS, 1% glutamax, 1% Pen/Strep, all by Gibco) with indicated ligands at 37 °C for 16 h. As the positive control, TNF-α (PeproTech, USA) at 100 ng mL^–1^ was used. After incubation, the cells were resuspended once in PBS and measured via flow cytometry (Attune NxT, Thermo Fisher).

### 4.13 Cytokine Release Assay

Raw 264.7 macrophages were cultured in complemented DMEM. A total of 10^5^ cells mL^–1^ were seeded in a 12-well plate and incubated overnight at 37 °C. The compounds were added (at concentrations of 1 μg mL^–1^) and the plate was incubated overnight at 37 °C. Supernatants were collected and stored at −80 °C until further use. For the quantitative measurement of cytokines, the levels were determined by ELISA using Murine TNF-α and Interleukin-6 Mini TMB ELISA development kits (PeproTech).

### 4.14 Complement Activation

AuNPs (2 μg mL^–1^, 100 μL) were incubated in commercially available normal human serum (100 μL) (Sigma-Aldrich, St. Louis, MO) for 1 h at 37 °C. PBS (10 mM, 100 μL) was used as the negative control. The mixture was then centrifuged to isolate AuNPs, and the serum-containing supernatant (100 μL) was used to analyze the concentration of the final product of complement activation, SC5b-9, induced by AuNPs of different configurations using an ELISA kit, following the procedure provided by the kit (Human TCC C5b-9, Biosite).

### 4.15 Toxicity Studies

Raw macrophages were cultures at 10^4^ cells/well for 24 h at 37 °C. The next day, the cells were cultured with the respective compounds (1 μg mL^–1^) in 90 μL of medium (complete RPMI medium without phenol red) per well for the required time. After 24 h, 10 μL of the MTT reagent (5 mg mL^–1^ in PBS) was added to each well. After incubation of 2 h, 100 μL of the MTT solvent (isopropanol + 40 mM HCl) was added to each well. The plate was incubated overnight at RT and then the absorbance at 570 nm was measured with a plate reader. The experiment was performed twice and in triplicates.

## Supporting Information Available

Detailed compound characterization of the nanoparticles (Figures S1–S4); quantification of mannose (Figure S5); binding to Con A and the toxicity study (Figures S6 and S7); and the precise concentration of sugars (Table S1); the .fcs files for the flow cytometry-based experiments are also available from the authors upon request (PDF)
