# Supplementary material for: Synthetic Glyconanoparticles Modulate Innate Immunity but Not the Complement System
Source: ACS Appl Bio Mater. 2022 Apr 18;5(5):2185–92. doi: 10.1021/acsabm.2c00026 (PMC9115801; doi:10.1021/acsabm.2c00026)
Supplement: Supplementary file 1 — mt2c00026_si_001.pdf [file mt2c00026_si_001.pdf]

Supporting Information

Synthetic glyco-nanoparticles modulate innate immunity but not the complement system

Chandradhish Ghosh [1,a], Patricia Priegue[1, 2], Harin Leelayuwapan[1,b], Felix F. Fuchsberger[1,c],

*Christoph Rademacher[1,c], and Peter H. Seeberger[1,2]**

[1] Department of Biomolecular Systems, Max Planck Institute of Colloids and Interfaces, Am Mühlenberg 1, 14476 Potsdam, Germany

[2] Institute of Chemistry and Biochemistry, Freie Universität Berlin, Arnimallee 22, 14195 Berlin, Germany

*peter.seeberger@mpikg.mpg.de

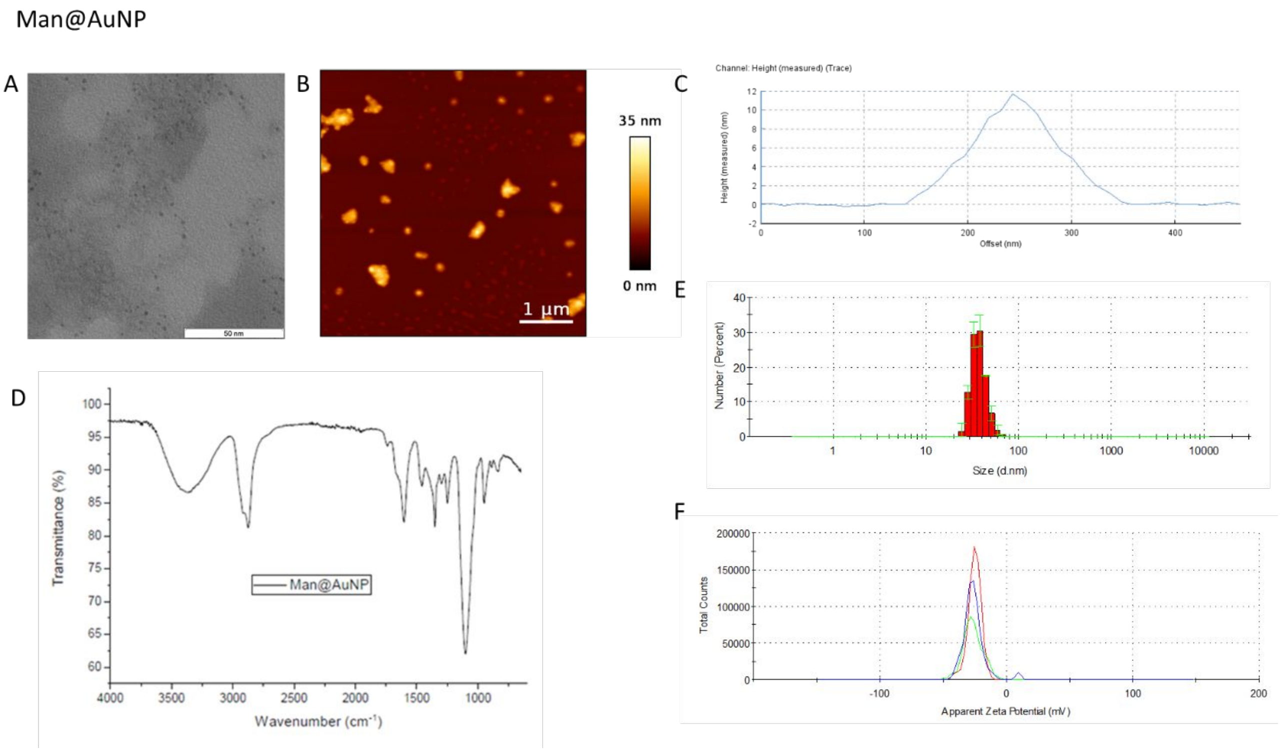

Figure S1. Characterization of Man@AuNP. (A) TEM images of Man@AuNP show the core structure of the gold nanoparticles to be <5 nm (Scale Bar 50 nm) (B) AFM image of Man@AuNP. (C) Height of the nanoparticle from AFM. (D) IR spectra of Man@AuNP shows prominent amide bond peaks at 1654 cm^{-1} and 1574 cm^{-1}. (E) Hydrodynamic radius of Man@AuNP. (F) Zeta potential of Man@AuNP is -30mV.

Man_2@AuNP

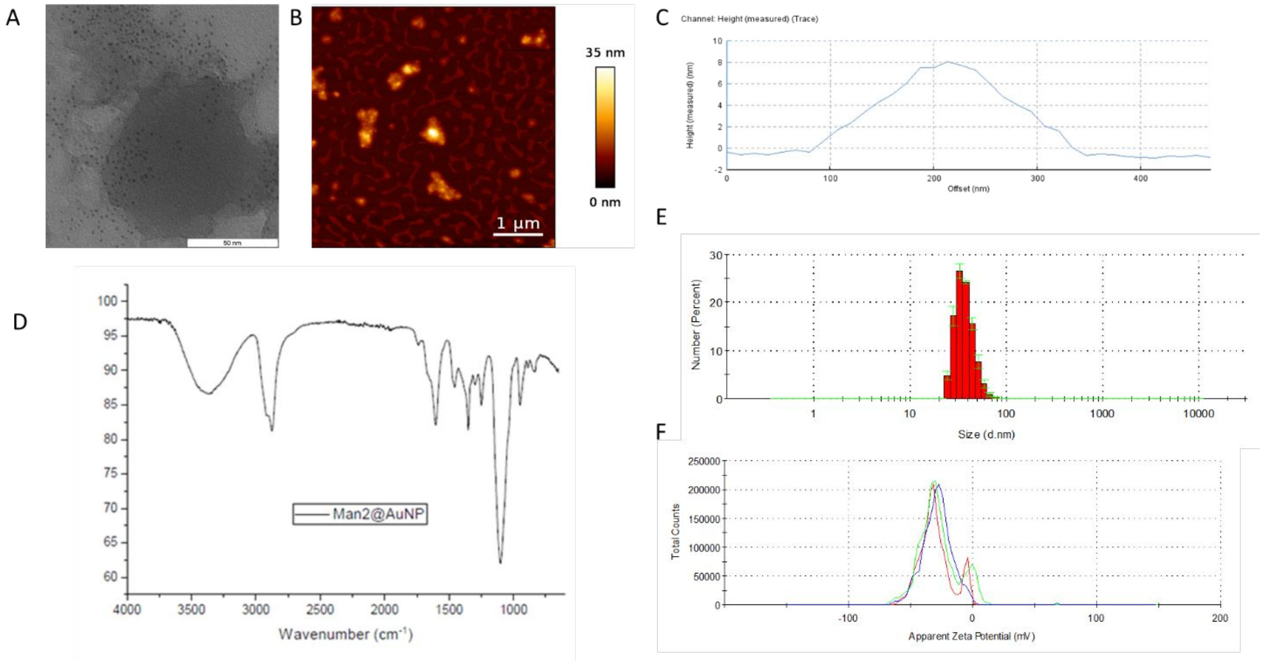

Figure S2. Characterization of nanoparticle Man_2@AuNP. (A) TEM images of Man_2@AuNP show the core structure of the gold nanoparticles to be <5 nm (Scale Bar 50 nm). (B) AFM image of Man_2@AuNP. (C) Height of the nanoparticle from AFM. (D) IR spectra of Man_2@AuNP shows prominent amide bond peaks at 1654 cm^{-1} and 1574 cm^{-1}. (E) Hydrodynamic radius of Man_2@AuNP. (F) Zeta potential of Man_2@AuNP is -30mV.

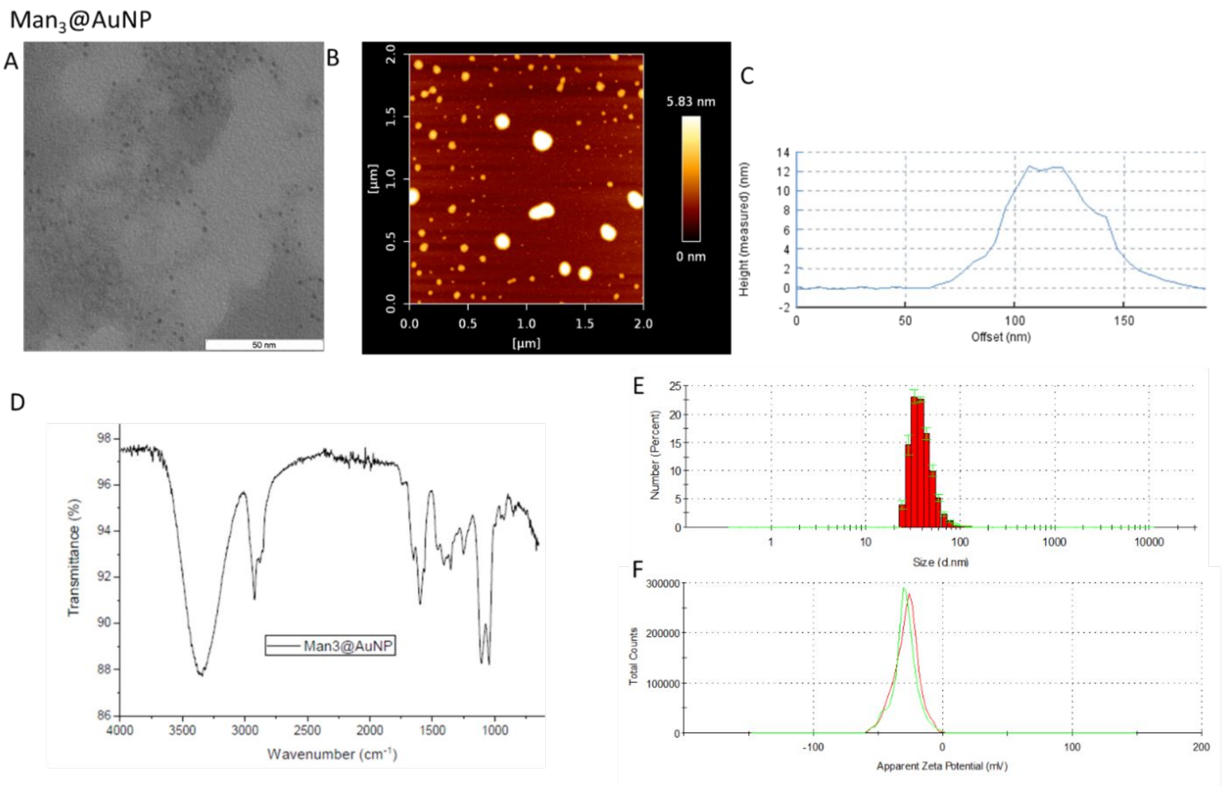

Figure S3. Characterization of representative nanoparticle, Man_3@AuNP. (A) TEM images of Man_3@AuNP show the core structure of the gold nanoparticles to be <5 nm (Scale Bar 50 nm). (B) AFM image of Man_3@AuNP. (C) Height of the nanoparticle from AFM. (D) IR spectra of Man_3@AuNP shows prominent amide bond peaks at 1654 cm^{-1} and 1574 cm^{-1}. (E) Hydrodynamic radius of Man_3@AuNP. (F) Zeta potential of Man_3@AuNP is -30mV.

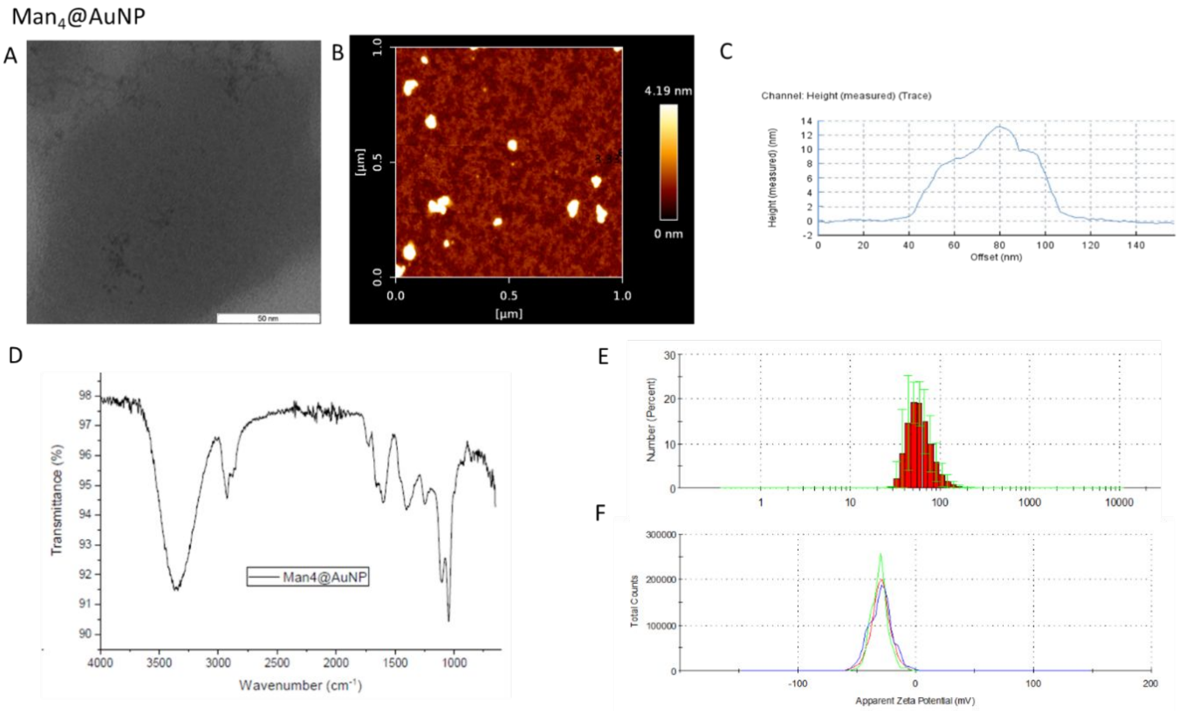

Figure S4. Characterization of nanoparticle Man$_4$@AuNP. (A) TEM images of Man$_4$@AuNP show the core structure of the gold nanoparticles to be <5 nm (Scale Bar 50 nm) (B) AFM image of Man$_4$@AuNP. (C) Height of the nanoparticle from AFM. (D) IR spectra of Man@AuNP shows prominent amide bond peaks at 1654 cm^{-1} and 1574 cm^{-1} that are absent in case AuNP. (E) Hydrodynamic radius of Man$_4$@AuNP. (F) Zeta potential of Man@AuNP is -30mV.

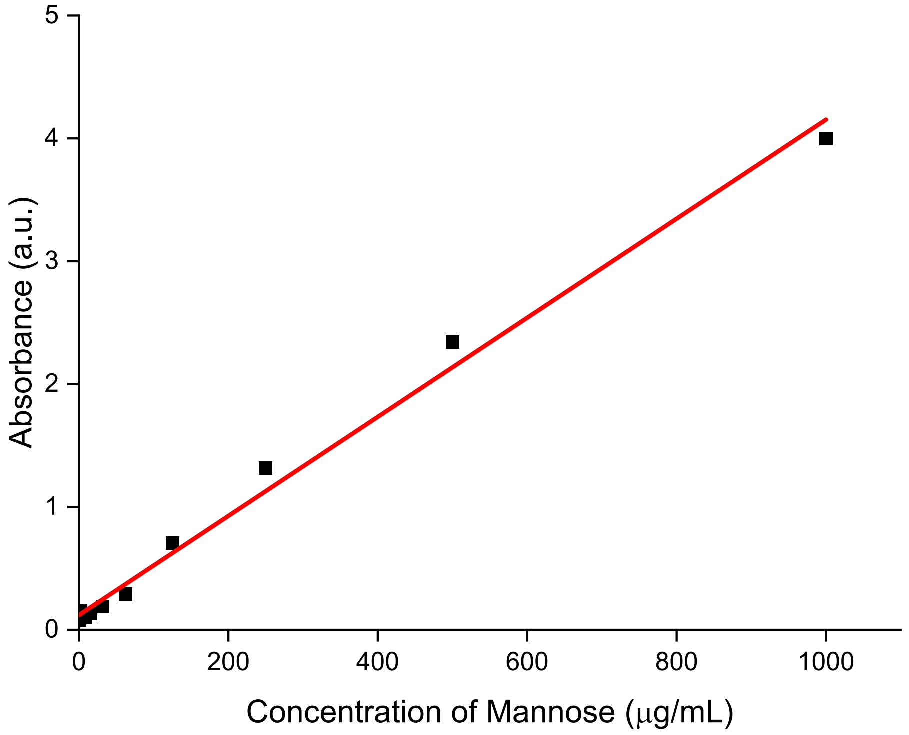

Figure S5. Standard curve for mannose quantification. Anthrone reacts with mannose in the presence of sulfuric acid, to form a colored compound that can be detected by absorption spectroscopy. The reaction is concentration dependent and can be monitored by measuring the absorbance at 620 nm.

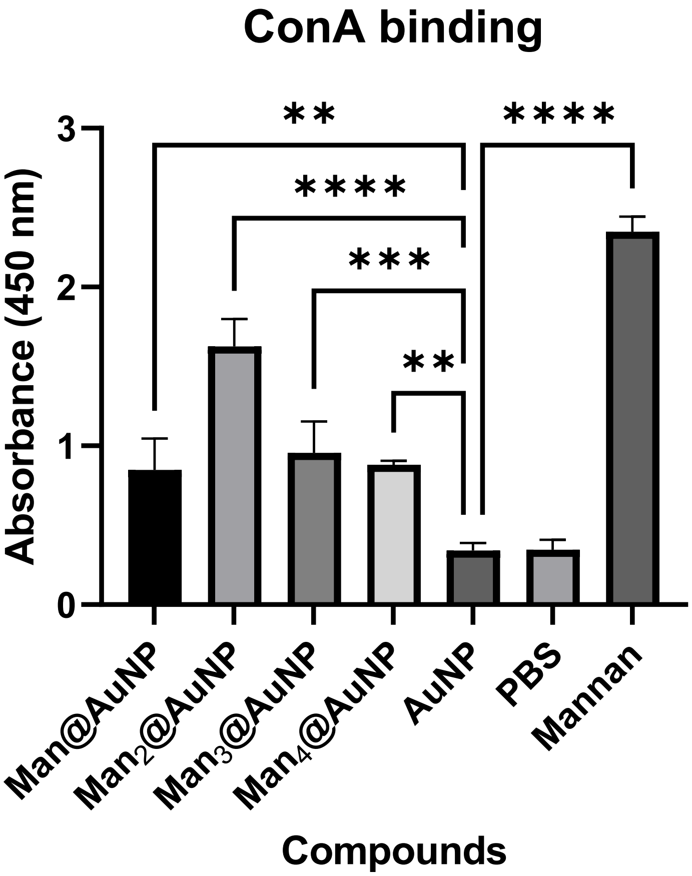

Figure S6. Binding to Concanavalin A. Mannan is used as a positive control while PBS is a negative control. All mannose conjugated nanoparticles showed a significant increase in binding to Con A in comparison to the naked nanoparticles. *, $p < 0.05$; **, $p < 0.01$,***, $p < 0.001$ and ****, $p < 0.0001$.

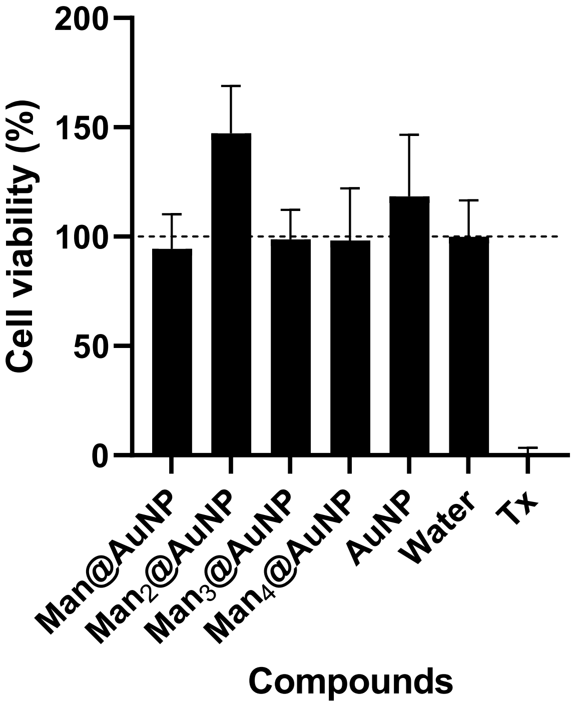

Figure S7. Toxicity study. Triton X is used as a positive control while water is used as a negative control. No toxicity was observed for any of the mannose conjugated nanoparticles.

Table S1. Concentration of sugars conjugated to nanoparticles as obtained from standard curve and also of the corresponding concentration of gold.

Nanoparticle	Concentration of mannose (μg mL^{-1})	Concentration of Gold (μg mL^{-1})
Man@AuNP	16.8	15

Man_2@AuNP	34.5	16
Man_3@AuNP	20.5	11
Man_4@AuNP	29.7	7
